# Effects of UVB-induced oxidative stress on protein expression and specific protein oxidation in normal human epithelial keratinocytes: a proteomic approach

**DOI:** 10.1186/1477-5956-8-13

**Published:** 2010-03-18

**Authors:** Marzia Perluigi, Fabio Di Domenico, Carla Blarzino, Cesira Foppoli, Chiara Cini, Alessandra Giorgi, Caterina Grillo, Federico De Marco, David A Butterfield, Maria E Schininà, Raffaella Coccia

**Affiliations:** 1 Department of Biochemical Sciences, "Sapienza" University of Rome - P.le A. Moro, 5 - 00185 Rome, Italy; 2 CNR Institute of Molecular Biology and Pathology - P.le A. Moro, 5 - 00185 Rome, Italy; 3 Laboratory of Virology, IFO - Regina Elena National Cancer Institute - V. Messi d'Oro, 156 - 00156 Rome, Italy; 4 Department of Chemistry, Center of Membrane Science, and Sanders-Brown Center on Aging, University of Kentucky, Lexington, KY 40506, USA

## Abstract

**Background:**

The UVB component of solar ultraviolet irradiation is one of the major risk factors for the development of skin cancer in humans. UVB exposure elicits an increased generation of reactive oxygen species (ROS), which are responsible for oxidative damage to proteins, DNA, RNA and lipids. In order to examine the biological impact of UVB irradiation on skin cells, we used a parallel proteomics approach to analyze the protein expression profile and to identify oxidatively modified proteins in normal human epithelial keratinocytes.

**Results:**

The expression levels of fifteen proteins - involved in maintaining the cytoskeleton integrity, removal of damaged proteins and heat shock response - were differentially regulated in UVB-exposed cells, indicating that an appropriate response is developed in order to counteract/neutralize the toxic effects of UVB-raised ROS. On the other side, the redox proteomics approach revealed that seven proteins - involved in cellular adhesion, cell-cell interaction and protein folding - were selectively oxidized.

**Conclusions:**

Despite a wide and well orchestrated cellular response, a relevant oxidation of specific proteins concomitantly occurs in UVB-irradiated human epithelial Keratinocytes. These modified (i.e. likely dysfunctional) proteins might result in cell homeostasis impairment and therefore eventually promote cellular degeneration, senescence or carcinogenesis.

## Background

The skin is the largest organ of the human body. It provides a major anatomical barrier between the internal and external environment. The body is constantly exposed to an array of chemical and physical exogenous pollutants. The outermost layer of the skin is composed predominantly by keratinocytes that provide a barrier between the host and the environment. Keratinocytes are continuously exposed to UV irradiation, which is able to induce a dramatic surge of biological events such as sunburn, inflammation, cellular/tissue injury, cell death, and skin cancer. Although UVB (290-320 nm) represents only 4% of the total solar UV radiation, it is responsible for the development of skin cancer in humans such as melanoma as well as non melanoma skin cancer [1].

Increasing evidence indicates that the UVB response in the skin is a complex and multifaceted biological process. The UVB signal transduction originates at multiple intracellular sites and the cross talk between dedicated molecular mediators acting within a complex signal network determines the fate of a UVB damaged cell. Even if very little is known about the original signalling mechanisms that trigger a UVB response in keratinocytes, it is well established that the detrimental effects of this type of radiation are associated with the formation of reactive oxygen species (ROS) [2,3].

ROS are formed and degraded by all aerobic organisms and are known to play a dual role in biological systems resulting either in beneficial or harmful effects. Beneficial effects involve physiological roles in cellular responses to noxious agents, for example in the defence against infections, and in the function of a number of cellular signalling systems [4,5]. Several cytokines, growth factors, hormones, and neurotransmitters use ROS as secondary messengers in the intracellular signal transduction [6]. Conversely, at high concentrations due to their high reactivity ROS are prone to cause damage and are thereby potentially toxic, mutagenic or carcinogenic [7,8]. All major groups of bio-molecules can be damaged by ROS action, undergoing structural and functional modifications.

Proteins, due to a combination of their UV absorption characteristics and their abundance in cells, are primary targets of UV-mediated cellular damage. UV radiation can damage proteins by direct oxidation or by covalent binding of lipid peroxidation breakdown products, resulting in loss of protein function and/or enzymatic activity [9-11]. The ROS oxidative attack on proteins causes reversible and/or irreversible modifications, such as carbonylation, nitration, glycation, formation of adducts with lipid peroxidation products and protein-protein cross linking. These modifications determine structural, functional and stability changes, leading to loss of function, fragmentation, unfolding/misfolding, protein aggregation and degradation.

Since proteins are the effectors of cellular functions, we applied in the present study a proteomics analysis to obtain a picture of target proteins that are specifically altered by UVB-mediated oxidative stress (OS) in normal human epithelial keratinocytes (NHEK). We analyzed the protein expression profile and identified the oxidatively modified proteins of UVB-treated cells compared to control cells.

## Results

### Identification of differentially expressed proteins

**Figure 1:**
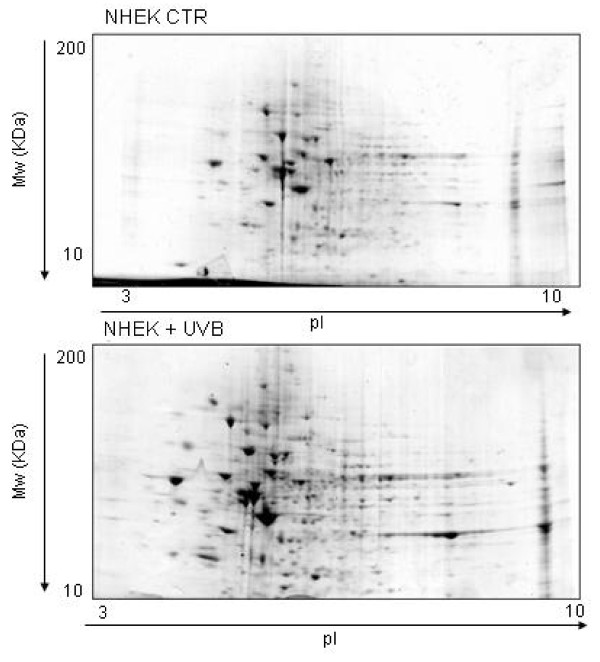
**2-DE proteomic maps of control (up) and UVB-irradiated (down) NHEK**. Protein (150 μg) extracts were analysed in first dimension (pH 3-10 linear IPG); second dimension was performed on slab gel (4-12% gradient SDS-PAGE). Protein detection was achieved using Biosafe Coomassie staining.

A proteomics approach was used to ascertain whether the UVB generated OS determined a qualitative and/or quantitative modification in the NHEK protein profiling. The UVB dosage chosen (20 J/m^2^) was able to induce intermediate cell damage without suppressing the cell response mechanisms (see Additional file 1). Total proteins extracted from UVB-irradiated and from control cells were subjected to two dimensional gel electrophoresis (2-DE). Software-assisted densitometric analysis of resolved gels allowed a comparison of the respective protein repertoires and the determination of quantitative modifications in the UVB-irradiated cells as compared to non-irradiated ones. Representative Coomassie-stained gels are shown in Figure 1, panel a and panel b.

The overall 2-DE pattern of UVB-treated cells and control cells were similar. However 15 spots were found to be differentially expressed with at least 1.5-fold increase or decrease (p < 0.059) compared to control cells. To assess reproducibility, the correlation coefficient among six replicated gels was calculated; the average r value of 0.9 indicated a high quality 2-DE gels and good reproducibility of culture and treatment conditions.

**Figure 2:**
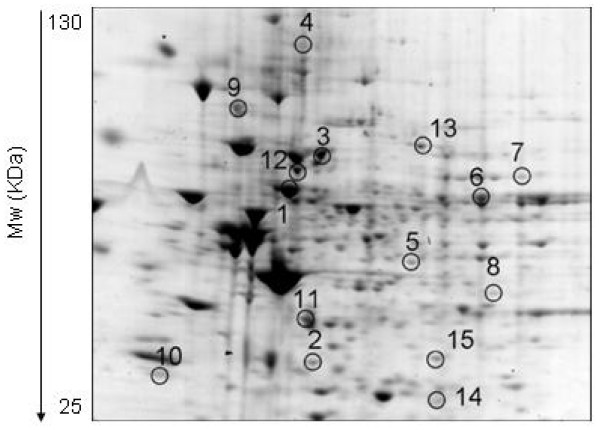
**Enlarged image of 2-DE proteomic map of UVB-irradiated NHEK**. The spots of proteins differentially expressed in irradiated cells respect to controls are represented by a progressive numeration (see Table 1).

Figure 2 shows an enlarged image of 2-DE gel of UVB-treated NHEK, with the differential expression of protein spots highlighted by circles and numbers.

The spots of differentially expressed proteins were excised from the gels, proteolysed and subjected to MS analysis. The database search with data deriving from Peptide Mass Fingerprint MALDI-ToF experiments allowed the identification of the spots. The list of the identified proteins is reported in Table 1, together with their quantitative variations, expressed as fold compared to control. 12 proteins were found to have higher abundance levels in UVB-irradiated NHEK compared to the control group, while 3 proteins were found to have a lower abundance level. All listed proteins included in Table 1 had p-values = 0.05, good sequence coverage, significant protein scores and similar observed and calculated molecular weights and isoelectric points.

**Table 1:** UVB-induced or suppressed proteins identified by mass spectrometry.

Spot n°	Protein name	**Fold**^a^	Theorethical Mw/pI	Sequence Coverage %	Protein Score	P value
1	HSP 60	1.45	61187/5.70	31	195	<0.05

2	Prohibitin	29.3	29843/5.57	50	177	<0.05

3	HSP 70	1.9	73920/5.87	50	281	<0.05

4	Integrin alpha-3	9.1	119820/6.60	8	117	<0.05

5	Ornithine aminotransferase	5.2	48846/6.57	32	163	<0.05

6	Cytokeratin 5	9.1	62568/7.59	27	228	<0.05

7	Phosphoenolpyruvate carboxykinase	3.9	71447/7.56	15	87	<0.05

8	26S proteasome subunit 7	4.4	37060/6.29	28	71	<0.05

9	GRP 78	0.05	72402/5.07	15	74	<0.05

10	Proteasome subunit alpha type-5	2.2	26565/4.74	39	111	<0.05

11	Actin	0.05	42052/5.29	30	102	<0.05

12	HSC 71	0.17	71082/5.37	38	179	<0.05

13	Serotransferrin precursor	3.1	79280/6.81	14	128	<0.05

14	Proteasome subunit alpha type-6	10.6	27838/6.34	31	131	<0.05

15	Proteasome subunit alpha type-1	2.6	29822/6.15	26	86	<0.05

The peptide mixtures obtained from trypsin treated protein spots, were analyzed by MALDI-ToF-MS and the relative mass lists used for identification by the Mascot program (Matrix Science).

### Specific protein carbonyl level

**Figure 3:**
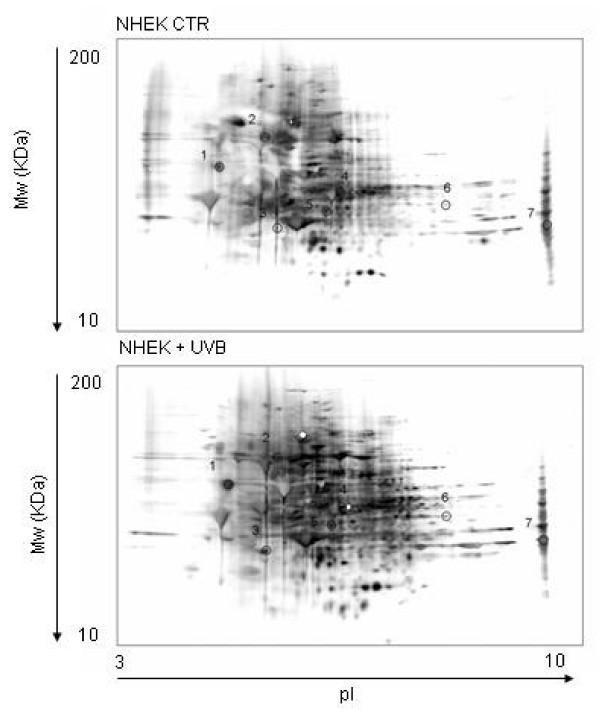
**Two-dimensional carbonyl immunoblots from control (up) or UVB-irradiated (down) NHEK**. Positions of the seven identified proteins are shown on the blots. The spots are represented by a progressive numeration (see Table 2).

Carbonylation is the most widely studied oxidative modification of proteins because of its ease in detection by the Western blot. Indeed, the protein-bound carbonyl groups upon reaction with DNPH generate stable protein-hydrazone complexes which are then easily detected by specific antibodies. The specific carbonylation level of each single spot was evaluated through the ratio between the carbonyl level of a protein spot on the nitrocellulose membrane and the protein level of its corresponding protein spot in the gel - assessed by Bio-Safe Coomassie staining and image analysis - and expressed as carbonyl level per unit of protein. In Figure 3 two-dimensional carbonyl immunoblots from control and UVB-treated NHEK are shown. 7 proteins resulted to be significantly more oxidized in irradiated cells compared to control cells. Table 2 illustrates the list of proteins that were successfully identified by the mass spectrometry, along with protein scores, sequence coverage, pI, Mw values and the increase of specific carbonyl levels.

**Table 2:** Proteins with increased oxidation after UVB irradiation of NHEK cells.

Spot n°	Protein name	Fold	Theorethical mw/pI	Sequence Coverage %	Protein Score	P value
1	Glucosidase 2 β subunit	4.2	60357/4.33	16	131	<0.002

2	GRP 78	4.5	72402/5.07	12	80	<0.02

3	Heterogeneous nuclear ribonucleoproteins C1/C2	1.55	33707/4.95	30	67	<0.04

4	Protein disulfide-isomerase A3	4.2	57146/5.98	42	226	<0.002

5	Actin-related protein 3	11.6	47797/5.61	19	110	<0.015

6	α-enolase	3.4	47481/7.01	31	112	<0.02

7	Annexin 2	5.1	36201/8.57	56	104	<0.02

For each protein the carbonyl immunoreactivity/protein expression values were averaged (n = 6) and expressed as fold increase in irradiated cells compared to control.

### Validation of identified proteins

To verify the proteomics and redox proteomics results, validation studies on protein up- or down-regulation and protein carbonylation were performed.

**Figure 4:**
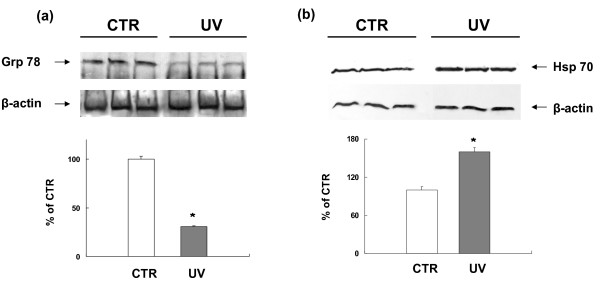
**Western blot for validation of identified proteins and their modulation in UVB-treated NHEK**. Total proteins (40 μg) extracted from irradiated and control cells were loaded onto a 12% SDS-PAGE gel, blotted onto nitrocellulose membrane and challenged with specific GRP78 and HSP70 antibodies. Western blot analysis confirmed proteomic results of decreased levels of GRP78 (a) and increased levels of HSP70 (b) in UVB-treated NHEK cells compared with control cells. Immunoblots were scanned by densitometry and all values were normalized to β-actin. Densitometric values are given as percentage of CTR values (n = 3) and represent the mean ± SEM of three independent experiments. * p < 0.001 vs CTR.

The modulation of protein expression level was validated by WB analysis in the case of glucose-regulated protein 78 (GRP78) and HSP70. The results are shown in Figure 4, where an increase of HSP70 and a parallel decrease of GRP78 can be seen in irradiated cells, thus confirming the same behaviour detected by proteomics, i.e. GRP78 down-regulation and HSP70 up-regulation.

**Figure 5:**
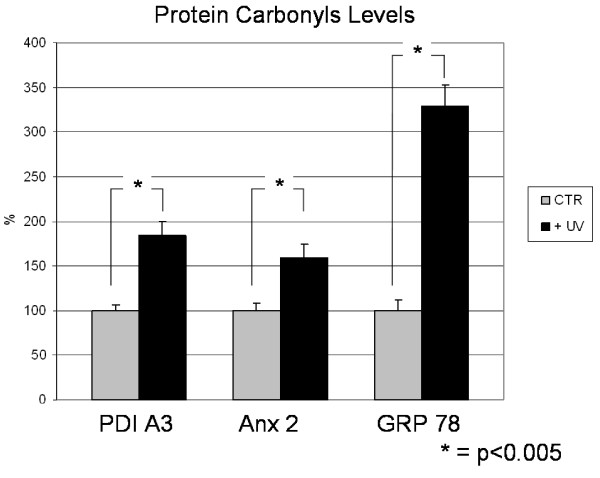
**Protein carbonyl levels of PDI A3, Anx2 and GRP78**. Data represent the alteration of the protein carbonyl levels in UV-treated NHEK cells compared to control cells using traditional immunochemical detection. Error bars indicate S.E.M for 3 samples in each group. Measured values are normalized with the mean of the control cells. *P < 0.005.

Redox proteomics results were validated by WB immunochemical detection of carbonylated proteins. In Figure 5, the carbonyl levels of protein disulfide isomerase A3 (PDI A3), Annexin 2 (Anx2) and GRP78 in UVB-treated cells were respectively about 180%, 160% and 320% in comparison to those of control cells, thus qualitatively confirming the redox proteomics findings.

The increase of carbonyl levels of PDI A3, Anx2 and GRP78 in UVB-treated cells were more robust when detected by the proteomics method. The differences in the magnitude of fold changes of carbonyl levels between the two techniques are likely because proteomics measures the carbonyl level per unit of protein, whereas WB measures the carbonyl level of the total protein.

### In vivo PDI A3 redox status

To evaluate the redox status of PDI A3 in NHEK cells, before and after treatment with UVB radiation, the cells were treated with the membrane permeable alkylating agent NEM to prevent disulfide exchange and freeze redox status. Then, cell lysates were treated with a second larger alkylating agent (AMS) that causes a shift in mobility when the protein is separated by SDS-PAGE. NEM alkylation performed on intact cells prevents AMS modification of free thiol residues present in the proteins. The second step in the alkylation was carried out after treating cell lysates with a thiol reducing agent that allows the modification of protein thiol residues present as disulfide bonds in intact cells. Thus the oxidized form of a protein can be resolved from the reduced one by its decreased electrophoretic mobility.

**Figure 6:**
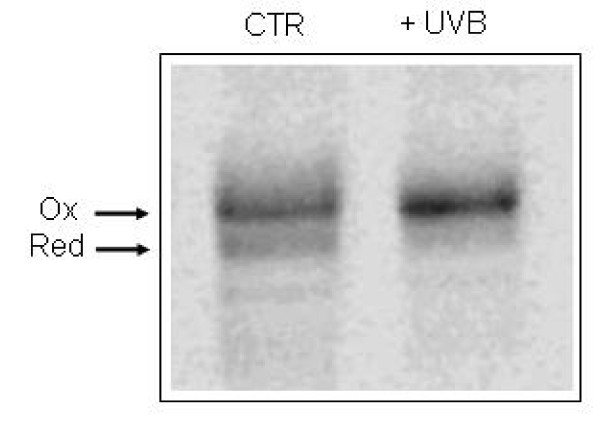
**PDI A3 redox status**. The redox status of PDI A3 was determined by sequential alkylation with NEM and AMS, SDS-PAGE and Western blotting with mouse anti-PDI A3 antibody. Protein bands were revealed by ECL assay using peroxidase-conjugate anti-mouse antibody and analyzed with a Kodak Image station 2400R equipped with a CCD. Lane 1: control, lane 2: UVB-irradiated NHEK. Upper band: oxidized form; lower band: reduced form.

Our results showed that in control cells PDI A3 was present both in oxidized and reduced form. Accordingly, WB analysis with anti-PDI A3 antibody revealed the presence of two bands related to oxidized and reduced forms of PDI A3 in NHEK untreated cells (Figure 6 lane 1). The treatment of cells with UVB radiation resulted in a significant increase in the oxidized form of PDI A3 as revealed by mobility shift (Figure 6 lane 2).

## Discussion

Exposure to UV radiation is the main risk factor for developing skin cancer [12]. The description of molecular alteration associated with UV exposure is a fundamental step toward a deeper understanding of carcinogenesis. The most immediate effect of UV interaction with living matter is the generation of reactive oxygen and nitrogen species (ROS and RNS). These highly reactive intermediates have the potential to attack and modify virtually any molecular component of the cell and are therefore considered the ultimate pathogenetic factor for senescent decay and degenerative disorders. Keratinocytes are the elective component of skin and mucosae and because of their physiologic role they are continuously exposed to UV irradiation. In the current study, we used a parallel proteomics and redox proteomics approach to identify those proteins which are differentially expressed as well as those specifically oxidized in normal human epithelial keratinocytes (NHEK) upon UVB-irradiation. This work ideally extends to NHEK a similar analysis previously performed on transformed keratinocytes [13].

Our results showed that in UVB-exposed cells 12 proteins were up-regulated, 3 proteins appeared down-regulated and 7 proteins were specifically oxidized. A number of defense and stress-related pathways were affected, including chaperones function, cell adhesion, cytoskeleton maintenance, misfolded proteins removal, cell growth and tumor suppression.

Following a toxic stimulus one of the most obvious effects is the accumulation of damaged and misfolded proteins. These have to be removed to avoid the deregulation or the suppression of related pathways. In this frame it is not surprising to find an increased expression of four subunits of the proteasome system, namely 26S subunit 7, subunit alpha type-1, type-5 and type-6. Proteasome is the major proteolytic system involved in the removal of abnormal and oxidatively damaged proteins [14]. Several studies reported a decrease of the proteasome activity and content in a number of degenerative and possibly neoplastic conditions [15,16] resulting in the accumulation of modified proteins that are able to prime deregulated functions. The up-regulation of the proteasome system in NHEK exposed to UVB irradiation suggests an appropriate response of these cells consistent with an increased activity of damaged proteins removal in order to counteract the toxic effect of oxidative agent. Indeed, cells exposed to OS are particularly dependent on the proteasome activity for removal of oxidized proteins which have been reported to be highly sensitive to proteasome degradation [14,17].

The findings of increased expression of both HSP60 and HSP70 underscore another cytoprotection mechanism active in NHEK. Heath Shock Proteins (HSPs) are a highly conserved system involved in protein misfolding prevention and repair [18]. They are induced by a broad spectrum of stresses and their role in directing defence mechanisms within cell fighting has been found in several stress conditions. Most HSPs are molecular chaperones sensing unfolded proteins and mediating their refolding, transport and interaction. Thereby, HSPs ensure maintenance of homeostasis and help cells to regain equilibrium following a perturbation. Recent evidence suggests that HSPs, by decreasing intracellular ROS in a glutathione-dependent way, enhance cell survival to OS [19].

In addition, our proteomic analysis indicated that UVB increased the expression of both prohibitin and alpha-3 integrin in the irradiated cells. Prohibitin is correlated with the process of senescence and is associated with antiproliferative activity in mammalian cells [20]. Therefore a role as tumour suppressor has been postulated for this growth negative regulator factor [21]. Integrin is a family of cell-surface receptors that couple the extracellular matrix outside a cell to the cytoskeleton inside the cell and transmit signals bidirectional across the plasma membrane by undergoing large-scale structural rearrangements. Mediating the cell-cell interaction during adhesion [22], they play an essential role in creating tight intercellular association. By regulating cell-cell and cell-matrix contacts, integrins participate in a wide-range of biological interactions and influence several important biologic processes, including growth, development, and differentiation. In particular, alpha 3-beta 1 integrin is necessary and sufficient for maximal keratinocyte survival on laminin-5 [23] and, since the invasive growth of various types of carcinomas is associated with a generalized decreased expression of integrin alpha subunits [24], a negative correlation between this protein and cancer growth can be expected. Because of the above reported findings, the concomitant increase of alpha-3 integrin and prohibitin expressions in NHEK indicates a homeostatic response of the cell to the potentially carcinogenic UVB stimulus with an up-regulation of two proteins with anti-proliferative and anti-invasive activities.

Among the identified proteins, our results showed a consistent increase of cytokeratin 5 expression after UVB irradiation. Cytokeratins belong to a large family of intermediate filaments that are important components of cytoskeleton of epithelial cells [25]. They can interact tightly with the plasma membrane, particularly at sites of cell-cell and cell-matrix adhesion at desmosomes and hemidesmosomes, multiprotein complexes forming a continuous link which secures the attachment of the basal keratinocytes to the underlying basement membrane.

Both cytokeratins and integrins have a crucial importance in the maintenance of epithelium structure and integrity. Because proper keratin gene expression and filament organization are absolutely necessary for normal functioning of the skin [26] and integrins are the main way by which cells both bind to and respond to their environment [27], the up-regulation of these classes of proteins could represent a mechanism for maintenance of functional properties of the epidermis.

Taken together, our results outline a consistent pattern indicating the ability of keratinocytes to counteract the harmful effects of UVB-induced oxidative stress. These results are in agreement with our previous report [28] showing that NHEK are considerably resistant to UVB irradiation, whose effect was rather mild and consisted in a moderate inhibition of proliferation, a mild reduction of mitochondrial activity, an appropriate induction of detoxifying and antioxidant enzymes while only a moderate induction of apoptotic cell death occurred.

Proteomics can also be utilized to analyze the post-translational modifications that regulate protein functions [29]. Through the redox proteomics approach we found 7 proteins with increased levels of carbonylation, namely protein-disulfide isomerase A3 (PDI A3), glucose-regulated protein 78 (GRP 78), actin-related protein 3 (Arp-3), glucosidase II β subunit, Annexin 2 (Anx 2), alpha-enolase and heterogeneous nuclear ribonucleoproteins C1/C2. (hnRNP).

PDI A3, also known as ERp57 or GRP58, is a member of the protein-disulfide isomerases family. Mainly localized in the endoplasmic reticulum (ER), it is strictly redox sensitive and it is involved in the correct folding and disulfide bond rearrangement of misfolded glycoproteins [30] and in the folding of major histocompatibility complexes [31,32]. In addition, PDI A3 mediates the integrin-dependent cell adhesion [33] and it is also involved in cell-cell interaction, gene expression, actin filament polymerization and regulation of reception functions. The elevated level of carbonylation here reported is not surprising considering that because of the function of the redox sensitive protein, PDI A3 is an elective target of UVB dependent OS. The consequent structural, functional and stability modifications, reasonably associated with loss of function, may deregulate those pathways in which are involved proteins whose folding is controlled by PDI A3. Interestingly, similar pathogenic mechanisms have recently been described in the homeostatic unbalance of degenerative diseases such as sporadic Parkinson's and Alzheimer's diseases [34]. In both cases nitrosylation of cysteine residues in the active sites of PDI determines that the enzyme cannot function as a folding catalyst, thereby leading to the accumulation of unfolded/misfolded proteins and their consequent degradation through the proteasome pathway.

Moreover, UVB irradiation determined both down-regulation and oxidation of GRP 78. This protein, also known as HSPA5 or BiP, is a member of the HSP70 family of proteins which function as molecular chaperones by binding transiently to proteins traversing through the ER and facilitating their folding, assembly, and transport. During the ER stress response, GRP78 binds misfolded proteins and translocates through ER membranes for their proteasomal degradation [35]. Decreased functionality of GRP 78 contributes to the accumulation of misfolded proteins that, if not properly degraded, have the ability to form toxic aggregates inside the cell.

Arp-3 (Actin-related protein 3) is a component of the Arp2/3 complex that is related in sequence and structure to actin and that binds ATP. Arp2/3 complex is an activator of actin filament nucleation and branching [36] and several lines of evidence indicate that it is necessary for cell protrusive activity associated with cell migration and invasion. We found that Arp-3 was consistently oxidized following UVB irradiation. This may lead to incorrect assembly of actin filaments and consequent alteration of cytoskeleton organization.

An increased oxidation of glucosidase II subunit beta was also found. Glucosidase II is one of the early N-glycan processing enzymes and a major player in the glycoprotein folding quality control. It is an ER enzyme that cleaves sequentially the two innermost α-1,3-linked glucose residues from N-linked oligosaccharides on nascent glycoproteins. This processing allows the binding and release of monoglucosylated glycoproteins with calnexin and calreticulin, the lectin-like chaperones of the ER [37]. An increased carbonylation level of β-subunit following UVB irradiation could lead to a decrease of enzymatic activity, finally resulting in an impairment of glycoprotein folding.

Interestingly, we found that Anx2 showed a significant increase of carbonyl levels in UVB-irradiated NHEK cells compared with control cells. Annexins are a family of proteins that bind acidic phospholipids in the presence of Ca^2+^. Their interaction with biological membranes has led to the suggestion that these proteins may play a role in membrane trafficking events such as exocytosis, endocytosis and cell-cell adhesion [38,39]. Recent studies suggest that Anx2 is regulated by the cellular redox status [40]. Therefore, Anx2 is an oxidatively labile protein and represents a selective target of oxidative damage mediated by UVB irradiation. We found a similar increase of Anx2 oxidation in HPV-transformed keratinocytes upon UVB exposure [13].

HnRNP C1 and C2 are involved in DNA repair and it has been shown that they play a pivotal role in coordinating repair pathways following exposure to ionising radiation, through protein-protein interactions and transcript regulation of key repair and stress response mRNA [41].

Since the susceptibility of α-enolase to different conditions of OS is well documented by several authors [42,43], we propose that its oxidation following UVB irradiation can be regarded as a non-specific event.

Essentially, oxidatively modified proteins are either functionally inactive or deregulated [44]. These structural and functional oxidative modifications could compromise the ability of cells to regulate homeostasis and account for the risk of cellular damage following UVB irradiation.

## Conclusions

In this study the effect of a subtoxic dose of UVB on proteome of normal human epithelial keratinocytes has been evaluated. In addition, the specific protein oxidation has been analyzed by the redox-proteomics approach. Among the proteins found up-regulated, of particular interest are those implicated in cell response to oxidative stress, i.e. HSPs and proteasome. On the other hand, proteins involved in protein folding, such as GRP78 and PDI, were found more oxidized in irradiated cells.

In conclusion, our results outline the ability of NHEK to activate some stress response pathways consistent with a cell protection response. However it is important to highlight that this thinly regulated cellular homeostasis may be overwhelmed by the consistent oxidation of target proteins. Further studies are needed to identify which molecular mechanisms could alter the balance between defence systems and accumulating oxidative damage causing the shift towards a pathologic condition.

## Materials and methods

### Cell cultures and UVB irradiation

Normal human epithelial keratinocytes (NHEK) were obtained from children's foreskins kindly donated by patients attending the general surgery division at the Ospedale S. Pertini (Rome, Italy), whose parents had released a full informed consent. NHEK were isolated and grown according to standard procedures [45] with minor modifications. Briefly, after scraping away excess fat and subcutaneous tissue, the foreskins were floated in 0.25% trypsin solution at 4°C overnight. The epidermis was then lifted off and placed in 10 ml of 0.5% trypsin-EDTA (Invitrogen Life Technologies, s.r.l. San Giuliano Milanese, Italy) at 37°C for 1 h under continuous mild stirring. Trypsin was neutralised by soybean trypsin inhibitor, the cell suspension was pelletted for 10 min at 200 × g, washed twice in phosphate buffered saline (PBS) and cultivated in keratinocytes serum free medium (K-SFM) (Invitrogen). Cells were maintained in a humidified incubator with 5% CO_2 _at 37°C and passaged twice a week at such a density they never reached 80% confluency. To avoid bias of senescence modification in cell metabolism, cells between third and eighth passages were used in the present study. For UVB treatment, cells were plated in a 100 mm/petri dish at a density of 80,000 cells/cm^2 ^(i.e. 50% confluency). Immediately before irradiation, the medium was removed and the monolayers were exposed to UVB in a home-made irradiation hood. To prevent overheating of monolayer, dishes were seated on a water bath at 37°C during the whole irradiation. UVB source was provided by a bank of Sankyo Denki G15T8E fluorescent tubes emitting 270-320 nm wavelength with a peak at 313 nm. The energy actually incident onto the working area was measured by a UVX Radiometer (UVP Inc., Upland, CA) and expressed in J/m^2^. The UV dosage of 20 J/m^2 ^(8 second exposure time) was chosen according to the results obtained in our previous studies [13] Such a dose was able to induce intermediate cell damage without suppressing the cell response mechanisms (see Additional file 1). After UV exposure, fresh medium was added and the cultures were further incubated. For negative control, cultures were decanted and placed in the irradiation chamber while keeping the UVB lamps switched off. Five hours after irradiation, cells were washed twice with ice cold PBS, scraped with a rubber policeman and pelletted.

### Sample preparation

Cell pellets were lysated in 10 mM HEPES buffer (pH 7.4) containing 137 mM NaCl, 4.6 mM KCl, 1.1 mM KH_2_PO_4_, 0.1 mM EDTA, and 0.6 mM MgSO_4 _as well as proteinase inhibitors: leupeptin (0.5 mg/ml), pepstatin (0.7 μg/ml), type II S soybean trypsin inhibitor (0.5 μg/ml), and PMSF (40 μg/ml). Cell lysates were centrifuged at 14,000 × g for 10 min to remove debris. Protein concentration in the supernatant was determined by using the Coomassie (Bradford) Protein Assay (Pierce, Rockford, IL, USA).

### Two-dimensional gel electrophoresis (2-DE)

The analysis was performed as previously described [13]. Sample volumes equivalent to 150 μg proteins were precipitated by adding of 100% ice-cold trichloroacetic acid (TCA) to a final concentration of 15% and placed on ice for 10 min. Precipitates were centrifuged at 15,000 × g for 2 min. The pellets were washed three times with 0.5 ml ethanol/ethyl acetate 1:1 solution. After centrifugation and washing, the samples were dissolved with 200 μL of rehydration buffer (8 M urea, 20 mM dithiothreitol, 2.0% (w/v) CHAPS, 0.2% Biolytes, 2 M thiourea and bromophenol blue).

For the first-dimension electrophoresis, 200 μL of sample solution were applied to a ReadyStrip™ IPG strip pH 3-10 (Bio-Rad Laboratories S.r.l., Segrate, Milano, Italy). The strips were soaked in the sample solution for 1 h to allow the uptake of proteins. The strip was then actively rehydrated in a Protean IEF Cell Apparatus (Bio-Rad) for 16 h at 50 V. The isoelectric focusing was performed at 300 V for 2 h linearly; 500 V for 2 h linearly; 1000 V for 2 h linearly, 8000 V for 8 h linearly and 8000 V for 10 h rapidly. All the processes above were carried out at room temperature. The focused IEF strips were stored at -80°C until second dimension electrophoresis was performed.

For second dimension electrophoresis, thawed strips were equilibrated for 10 min in 50 mM Tris-HCl (pH 6.8) containing 6 M urea, 1% (w/v) sodium dodecyl sulfate (SDS), 30% (v/v) glycerol, and 0.5% dithiothreitol, and then re-equilibrated for 15 min in the same buffer containing 4.5% iodacetamide in place of dithiothreitol. Linear Gradient (4-12%) Precast criterion XT gels (Bio-Rad) were used to perform second dimension electrophoresis. Precision Protein™ Standards (Bio-Rad) were run along with the sample at 200 V for 65 min.

For expression analysis, after electrophoresis the gels were incubated 20 min in fixing solution (7% acetic acid, 10% methanol), stained for 1 h in approximately 40 ml of Bio-Safe Coomassie Gel Stain (Bio-Rad) under continuous gentle agitation and destained overnight in deionized water.

### Western blot immunochemical detection of protein carbonyl levels

For the protein oxidation analysis, gels were transferred to nitrocellulose membrane (Bio-Rad) using Criterion Blotter apparatus (Bio-Rad) at 100 V for 1 h according to the supplier's instructions. The carbonyl levels were detected by post-Western blot derivatization of 2D nitrocellulose membrane [46]. Briefly, following the electroblotting procedure, the nitrocellulose membranes were equilibrated in 20% methanol for 5 min and then incubated in 2 N HCl for 5 min. Next, membranes were incubated in 0.5 mM 2,4-dinitrophenyl hydrazine (DNPH) solution for 5 min sharp. The membranes were washed three times in 2 N HCl and five times in 50% methanol (5 min each wash). The 2,4-dinitrophenyl hydrazone (DNP) adducts of the protein carbonyls were detected on the nitrocellulose sheet using a primary rabbit antibody (Millipore Corp., MA, USA) specific to DNP-protein adduct (1:100), followed by a secondary goat anti-rabbit IgG alkaline-phosphatase conjugated antibody (Sigma-Aldrich, Milano, Italy). The resultant stain was developed using 5-bromo-4-chloro-3-indolyl phosphate/nitro blue tetrazolium (BCIP/NBT) solution (SigmaFast tablets from Sigma).

### Image Analysis

The 12 gels (n = 6 controls and n = 6 UVB-treated cells) and 12 nitrocellulose blots were scanned and saved in TIF format using a Scanjet 3300C (Hewlett Packard). PDQuest 2D Analysis Software (version 7.2.0, Bio-Rad) was used for protein spot matching and analysis and to compare proteins and DNP immunoreactivity content between UV-treated and control cells. This software offers powerful comparative analysis and is specifically designed to analyze many gels or blots at once. Powerful automatching algorithms quickly and accurately match gels or blots and sophisticated statistical analysis tools identify experimentally significant spots. The principles of measuring intensity values by 2-D analysis software were similar to those of densitometric measurement. The average mode of background subtraction was used to normalize intensity values amount of protein (total protein on gel versus DNP-bound protein on the membrane) per spot. Once spots had been matched, images were manually edited to confirm proper spot detection and matching. The intensity of each protein spot was normalized as a percentage of total volume, corresponding to pixel intensity integrated over the area of each spot and divided by the sum of all spots in the gel to account for staining variability. Following manual editing and matching confirmation, average normalized spot volumes (pixel intensity over spot area) were compared between UVB-treated and control cells. Target candidates were identified as protein spots that changed at least 1.5-fold versus their specific control or alternatively that were either present or absent either in control or in experimental gel. Protein spots with greater than 50% internal variance were removed from the target list. Finally, remaining individual candidates were visually examined to ensure that the change was consistent in all gels.

After completion of spot matching, the normalized intensity of each protein spot from individual gels was compared between groups using statistical analysis. Statistical significance was assessed by a two-tailed Student's *t*-test, the method of statistical analysis most appropriate for proteomic analysis of small number of protein spots [47]. P values < 0.05 were considered significant for comparison between control and experimental data (UV-treated NHEK cells).

### Protein identification by mass spectrometry

Selected spots were manually excised from gels and submitted to trypsin proteolysis, as described by Mignogna et al. [48], with little difference. In short, after four destaining steps using 5% (30 min), 50% (2 times, 30 min each), and 100% (10 min) acetonitrile in 25 mM ammonium bicarbonate, about 165 ng of trypsin (modified porcine variant, Promega, Madison, WI, USA) were solubilised in 15 μl of a 25 mM ammonium bicarbonate digestion buffer and added to each vacuum-dried gel spot. Digestion was performed at 37°C overnight. The peptide mixtures were eluted directly onto an appropriate MALDI target plate with 1.3 μl of α-cyano-4-hydroxy-trans-cinnamic acid matrix solution (2 mg/ml) in 70% acetonitrile containing 0.1% TFA (v/v). MALDI-ToF MS analyses were performed in a Voyager-DE STR instrument (Applied Biosystems, Framingham, MA, USA) equipped with a 337 nm nitrogen laser and operating in reflector mode. Mass data were obtained by accumulating several spectra from laser shots with an accelerating voltage of 20 kV. All mass spectra were externally calibrated using a standard peptide mixture containing des-Arg-bradykinin (m/z 904.4681), angiotensin I (m/z 1296.6853), 1-17 (m/z 2093.0867), and 18-39 (m/z 2465.1989) adrenocorticotropic hormone fragments. Two tryptic autolytic peptides were also used for the internal calibration (m/z 842.5100 and 2807.3145). Several ion signals were submitted to fragmentation by post source decay (PSD). PSD fragment ion spectra were obtained after isolation of selected precursor ions using a timed ion selector (TIS), performing 10 steps of the reflectron voltage; for each individual step the voltage was decreased 25% with respect to the previous step. The individual segments were automatically stitched together. The PSD fragment ions were measured as isotopically averaged masses. Calibration was performed with PSD spectra of angiotensin.

The MS and MS/MS data were analysed by MoverZ program (v. 2002, http://bioinformatics.genomicsolutions.com), according to default parameters.

Identification by peptide mass fingerprint (PMF), with the monoisotopic mass list obtained from each spot, after exclusion of expected contaminant mass values by Peak Erazor program http://www.protein.sdu.dk/gpmaw/Help/PeakErazor/peakerazor.html, was performed using the Mascot search engine (v. 2.1) against SwissProt database (v. 52.2, entries 263525). Up to one missed cleavage, 50 ppm measurement tolerance, oxidation at methionine (variable modification) and carbamidomethyl cysteine (fixed modification) were considered. Post-translational modifications were not considered. Identifications were validated when the probability-based Mowse protein score was significant according to Mascot [49].

Identification by tandem mass spectrometry analyses was performed using the Mascot search program (Version 2.1) against human SwissProt database (v. 54.6×, 290484 sequences; 107100015 residues; date 2008/01/03), with mass tolerance of ± 0.5 Da for the precursor ions and ± 0.8 Da for the fragment ions, with carbamidomethyl cysteine as fixed modification. The expectation value (E-value) for accepting identification by MS/MS spectra was set to < 0.1, with a default significance threshold p < 0.05, that provides a 95% confidence level.

### Western blot analysis

For Western blot analysis a 40 μg aliquot of each protein sample was separated through a 12% SDS-PAGE and electroblotted (1 h at 100 V) to nitrocellulose membranes (Bio-Rad) using 25 mM Tris, 192 mM glycine and 20% (v/v) methanol. Equal protein loading was confirmed by 0.2% v/v Ponceau S in 7% acetic acid blot staining. Blotted membranes were blocked with 5% no-fat milk and challenged with appropriate primary antibodies, namely Anx2 mouse monoclonal IgG (Abnova GmbH, Heidelberg, Germany), GRP78 rat monoclonal IgG (Santa Cruz Biotech. Inc., Santa Cruz, CA, USA) and PDI A3 antibody (kindly provided by Prof. F. Altieri, Rome) for 1 h at room temperature. Unbound antibodies were removed by washing it twice with Tris-buffered saline containing 0.1% Tween 20, for 5 minutes. The membranes were then incubated with horseradish peroxidase-conjugated secondary antibody diluted 1:5000. Protein bands were visualized with ECL PlusTM (Amersham, NJ, USA) according to the manufacturer's protocol.

### Immunoprecipitation

The immunoprecipitation was performed as described by Lauderback et al. [50] Antibodies were added directly to cell lysates with IP Buffer (NaCl 0.15 M, NP-40 0.5%, Tris-HCl 50 mM pH 7.2, protease inhibitors) and the mixture was incubated on a rotary mixer overnight at 4°C. The antigen/antibody complexes were precipitated with protein-A-conjugated agarose beads if the antibodies were raised in rabbit or with protein G-conjugated agarose beads if the antibodies were raised in goat or mouse. Agarose beads were added in 50 μl aliquots from a stock of 300 mg/ml in PBS and mixed on a rotary mixer for 1 h at room temperature. Beads were then pelletted and washed three times with washing buffer (pH 8, 50 mM Tris HCl, 150 mM NaCl, 0.1% Tween 20). Proteins were eluted in IEF rehydration buffer followed by a 2D electrophoresis (Anx2) or in sample buffer for post-Western blot analysis (PDI A3, Anx2 and GRP78).

### Determination of redox status of Protein disulfide isomerase A3 (PDI A3)

To determine the *in vivo *redox status of PDI A3/ERp57, UVB-treated NHEK were subjected to thiols sequential alkylation with *N*-ethylmaleimide (NEM) and 4-acetamido-4'-maleimidylstilbene-2,2'-disulfonic acid (AMS), as described by Jessop and Bulleid [51] and modified according to Kim-Han and O'Malley [52]. In short, cells were incubated with 25 mM NEM to block free thiols and then lysed in 50 mM Tris-HCl pH 7.5, 150 mM NaCl, 2 mM EDTA, 1% Triton X-100 and protease inhibitor cocktail. Lysates were then treated with 2% SDS and 50 mM DTT in order to reduce all thiol residues, precipitated with 10% trichloroacetic acid and washed with 70% ice cold acetone. Finally, proteins were resuspended in 80 mM Tris-HCl pH 6.8, 2% SDS, protease inhibitor cocktail and 30 mM AMS in order to alkylate free thiol residues. Samples were separated by 10% SDS-PAGE and analyzed by Western blot with PDI A3 specific antibody [51,52]. AMS alkylated proteins (i.e. proteins with oxidised thiol groups) had a reduced electrophoretic mobility compared with non derivatized (reduced) proteins.

### Statistical analysis

Two-sided, Student's t-tests were used to analyze differences in protein levels between UVB-treated NHEK cell lysates and control untreated lysates. According to the exhaustive analysis of Maurer and Peters [47] the significance of carbonylation change of specific proteins was evaluated via nonparametric Mann-Whitney-Wilcoxon test. P < 0.05 was considered statistically significant.

## Competing interests

The authors declare that they have no competing interests.

## Authors' contributions

MP and FDD have made substantial contributions to conception and design of the experiments, image analysis and protein identification. CB carried out the 2-DE experiments, sample digestion an western blotting. AG and MES performed the MALDI-ToF acquisition and interpretation of data. CG carried out PDI determination. FDM contributed to experiments conception and was responsible for cell isolation and culture. CF, CC, DAB and RC participated in the data analysis, coordination and preparation of the final version of the manuscript. All authors have read and approved the final manuscript.

## Supplementary Material

Additional file 1 **Identification of UVB sub-toxic dose**. Data provided describe the UVB Dose-response and time-course tissue culture toxic effect; Description of methods, results, comments, figures and references are provided. Click here for file

## References

[B1] Attar M Lloyd JR Bickers DR Mukhtar H Malignant conversion of UV radiation and chemically induced mouse skin benign tumors by free-radical-generating compounds Carcinogenesis 1989 10 1841 1845 10.1093/carcin/10.10.1841 2507187

[B2] Paz ML González Maglio DH Weill FS Bustamante J Leoni J Mitochondrial dysfunction and cellular stress progression after ultraviolet B irradiation in human keratinocytes Photodermatol Photoimmunol Photomed 2008 24 115 122 10.1111/j.1600-0781.2008.00348.x 18477129

[B3] Kovacs D Raffa S Flori E Aspite N Briganti S Cardinali G Torrisi MR Picardo M Keratinocyte growth factor down-regulates intracellular ROS production induced by UVB J Dermatol Sci 2009 54 106 113 10.1016/j.jdermsci.2009.01.005 19250802

[B4] D'Autréaux B Toledano MB ROS as signalling molecules: mechanisms that generate specificity in ROS homeostasis Nat Rev Mol Cell Biol 2007 8 813 824 10.1038/nrm2256 17848967

[B5] Kamata H Hirata H Redox regulation of cellular signalling Cell Signal 1999 11 1 14 10.1016/S0898-6568(98)00037-0 10206339

[B6] Thannickal VJ Fanburg BL Reactive oxygen species in cell signaling Am J Physiol Lung Cell Mol Physiol 2000 279 L1005 1028 11076791 10.1152/ajplung.2000.279.6.L1005

[B7] Marnett LJ Oxyradicals and DNA damage Carcinogenesis 2000 21 361 370 10.1093/carcin/21.3.361 10688856

[B8] Toyokuni S Okamoto K Yodoi J Hiai H Persistent oxidative stress in cancer FEBS Lett 1995 358 1 3 10.1016/0014-5793(94)01368-B 7821417

[B9] Punnonen K Puntala A Jansén CT Ahotupa M UVB irradiation induces lipid peroxidation and reduces antioxidant enzyme activities in human keratinocytes in vitro Acta Derm Venereol 1991 71 239 242 1678228

[B10] Stadtman ER Berlett BS Reactive oxygen-mediated protein oxidation in aging and disease Drug Metab Rev 1998 30 225 243 10.3109/03602539808996310 9606602

[B11] Esterbauer H Schaur RJ Zollner H Chemistry and biochemistry of 4-hydroxynonenal, malonaldehyde and related aldehydes Free Radic Biol Med 1991 11 81 128 10.1016/0891-5849(91)90192-6 1937131

[B12] LeBlanc WG Vidal L Kirsner RS Lee DJ Caban-Martinez AJ McCollister KE Arheart KL Chung-Bridges K Christ S Clark J Lewis JE Davila EP Rouhani P Fleming LE Reported skin cancer screening of US adult workers J Am Acad Dermatol 2008 59 55 63 10.1016/j.jaad.2008.03.013 18436338 PMC3209702

[B13] Perluigi M Giorgi A Blarzino C De Marco F Foppoli C Di Domenico F Butterfield DA Schininà ME Cini C Coccia R Proteomics analysis of protein expression and specific protein oxidation in human papillomavirus transformed keratinocytes upon UVB irradiation J Cell Mol Med 2009 13 1809 1822 10.1111/j.1582-4934.2008.00465.x 19267883 PMC6512374

[B14] Jung T Grune T The proteasome and its role in the degradation of oxidized proteins IUBMB Life 2008 60 743 752 10.1002/iub.114 18636510

[B15] Dahlmann B Role of proteasomes in disease BMC Biochem 2007 8 Suppl 1 S3 10.1186/1471-2091-8-S1-S3 18047740 PMC2106367

[B16] Keller JN Hanni KB Markesbery WR Impaired proteasome function in Alzheimer's disease J Neurochem 2000 75 436 9 10.1046/j.1471-4159.2000.0750436.x 10854289

[B17] Petropoulos I Conconi M Wang X Hoenel B Brégégère F Milner Y Friguet B Increase of oxidatively modified protein is associated with a decrease of proteasome activity and content in aging epidermal cells J Gerontol A Biol Sci Med Sci 2000 55 B220 227 10819308 10.1093/gerona/55.5.b220

[B18] Timperio AM Egidi MG Zolla L Proteomics applied on plant abiotic stresses: Role of heat shock proteins (HSP) J Proteomics 2008 71 391 411 10.1016/j.jprot.2008.07.005 18718564

[B19] Arrigo AP Virot S Chaufour S Firdaus W Kretz-Remy C Diaz-Latoud C Hsp27 consolidates intracellular redox homeostasis by upholding glutathione in its reduced form and by decreasing iron intracellular levels Antioxid Redox Signal 2005 7 414 422 10.1089/ars.2005.7.414 15706088

[B20] McClung JK King RL Walker LS Danner DB Nuell MJ Stewart CA Dell'Orco RT Expression of prohibitin, an antiproliferative protein Exp Gerontol 1992 27 413 418 10.1016/0531-5565(92)90074-A 1459218

[B21] Nuell MJ Stewart DA Walker L Friedman V Wood CM Owens GA Smith JR Schneider EL Dell' Orco R Lumpkin CK Danner DB Mc-Clung JK Prohibitin, an evolutionarily conserved intracellular protein that blocks DNA synthesis in normal fibroblasts and HeLa cells Mol Cell Biol 1991 11 1372 1381 1996099 10.1128/mcb.11.3.1372-1381.1991 PMC369408

[B22] Carter WG Wayner EA Bouchard TS Kaur P The role of integrins α2 β1 and α3 β1 in cell-cell and cell-substrate adhesion of human epidermal cells J Cell Biol 1990 110 1387 1404 10.1083/jcb.110.4.1387 1691191 PMC2116091

[B23] Manohar A Shome SG Lamar J Stirling L Iyer V Pumiglia K DiPersio CM Alpha 3 beta 1 integrin promotes keratinocyte cell survival through activation of a MEK [?]/ERK [?] signaling pathway J Cell Sci 2004 117 4043 4054 10.1242/jcs.01277 15280429

[B24] Pignatelli M Hanby AM Stamp GW Low expression of β1, α2 and α3 subunits of VLA integrins in malignant mammary tumours J Pathol 1991 165 25 32 10.1002/path.1711650106 1659627

[B25] Moll R Franke WW Schiller DL Geiger B Krepler R The catalog of human cytokeratins: patterns of expression in normal epithelia, tumors and cultured cells Cell 1982 3 11 24 10.1016/0092-8674(82)90400-7 6186379

[B26] Rao KS Babu KK Gupta PD Keratins and skin disorders Cell Biol Int 1996 20 261 74 10.1006/cbir.1996.0029 8664850

[B27] Aplin AE Howe A Alahari SK Juliano RL Signal transduction and signal modulation by cell adhesion receptors: the role of integrins, cadherins, immunoglobulin-cell adhesion molecules, and selectins Pharmacol Rev 1998 50 197 263 9647866

[B28] De Marco F Perluigi M Foppoli C Blarzino C Cini C Coccia R Venuti A UVB irradiation down-regulates HPV-16 RNA expression: implications for malignant progression of transformed cells Virus Res 2007 130 249 259 10.1016/j.virusres.2007.06.018 17683822

[B29] Mann M Jensen ON Proteomic analysis of post-translational modifications Nat Biotechnol 2003 21 255 261 10.1038/nbt0303-255 12610572

[B30] Elliott JG Oliver JD High S The thiol-dependent reductase ERp57 interacts specifically with N-glycosylated integral membrane proteins J Biol Chem 1997 272 13849 13855 10.1074/jbc.272.21.13849 9153243

[B31] Antoniou AN Ford S Alphey M Osborne A Elliott T Powis SJ The oxidoreductase ERp57 efficiently reduces partially folded in preference to fully folded MHC class I molecules EMBO J 2002 21 2655 2663 10.1093/emboj/21.11.2655 12032078 PMC126025

[B32] Kang SJ Cresswell P Regulation of intracellular trafficking of human CD1d by association with MHC class II molecules EMBO J 2002 21 1650 1660 10.1093/emboj/21.7.1650 11927549 PMC125936

[B33] Lahav J Wijnen EM Hess O Hamaia SW Griffiths D Makris M Knight CG Essex DW Farndale RW Enzymatically catalyzed disulfide exchange is required for platelet adhesion to collagen via integrin a2h1 Blood 2003 102 2085 2092 10.1182/blood-2002-06-1646 12791669

[B34] Uebara T Nakamura T Yao D Shi ZQ Gu Z Ma Y Masliah E Nomura Y Lipton SA S-nitrosylated protein disulphide isomerase links protein misfolding to neurodegeneration Nature 2006 441 513 517 10.1038/nature04782 16724068

[B35] Falahatpisheh H Nanez A Montoya-Durango D Qian Y Tiffany-Castiglioni E Ramos KS Activation profiles of HSPA5 during the glomerular mesangial cell stress response to chemical injury Cell Stress Chaperones 2007 12 209 218 10.1379/CSC-259.1 17915553 PMC1971237

[B36] Millard TH Sharp SJ Machesky LM Signalling to actin assembly via the WASP (Wiskott-Aldrich syndrome protein)-family proteins and the Arp2/3 complex Biochem J 2004 380 1 17 10.1042/BJ20040176 15040784 PMC1224166

[B37] Pelletier MF Marcil A Sevigny G Jakob CA Tessier DC Chevet E Menard R Bergeron JJ Thomas DY The heterodimeric structure of glucosidase II is required for its activity, solubility, and localization in vivo Glycobiology 2000 10 815 827 10.1093/glycob/10.8.815 10929008

[B38] Waisman DM Annexin II tetramer: structure and function Mol Cell Biochem 1995 149-150 301 322 10.1007/BF01076592 8569746

[B39] Moss SE Annexins Trends Cell Biol 1997 7 87 89 10.1016/S0962-8924(96)10049-0 17708913

[B40] Tanaka T Akatsuka S Ozeki M Shirase TH Toyokuni S Redox regulation of Annexin 2 and its implications for oxidative stress-induced renal carcinogenesis and metastasis Oncogene 2004 23 3980 3989 10.1038/sj.onc.1207555 15048081

[B41] Haley B Paunesku T Protić M Woloschak GE Response of heterogeneous ribonuclear proteins (hnRNP) to ionising radiation and their involvement in DNA damage repair Int J Radiat Biol 2009 85 643 655 10.1080/09553000903009548 19579069 PMC2785495

[B42] Castegna A Aksenov M Thongboonkerd V Klein JB Pierce WM Booze R Markesbery WR Butterfield DA Proteomic identification of oxidatively modified proteins in Alzheimer's disease brain. Part II: dihydropyrimidinase-related protein 2, alpha-enolase and heat shock cognate 71 J Neurochem 2002 82 1524 1532 10.1046/j.1471-4159.2002.01103.x 12354300

[B43] Tamarit J Cabiscol E Ros J Identification of the major oxidatively damaged proteins in Escherichia coli cells exposed to oxidative stress J Biol Chem 1998 273 3027 3032 10.1074/jbc.273.5.3027 9446617

[B44] Levine RL Stadtman ER Oxidative modification of proteins during aging Exp Gerontol 2001 36 1495 1502 10.1016/S0531-5565(01)00135-8 11525872

[B45] Pirisi L Yasumoto S Feller M Doniger J Di Paolo JA Transformation of human fibroblasts and keratinocytes with human papillomavirus type 16 DNA J Virol 1987 61 1061 1066 2434663 10.1128/jvi.61.4.1061-1066.1987 PMC254063

[B46] Conrad CC Talent JM Malakowsky CA Gracy RW Post-Electrophoretic Identification of Oxidized Proteins Biol Proced Online 2000 2 39 45 10.1251/bpo17 12734585 PMC140127

[B47] Maurer HH Peters FT Toward high-throughput drug screening using mass spectrometry Ther Drug Monit 2005 27 686 688 10.1097/01.ftd.0000180224.19384.f0 16404794

[B48] Mignogna G Giorgi A Stefanelli P Neri A Colotti G Maras B Schininà ME Inventory of the proteins in Neisseria meningitidis serogroup B strain MC58 J Proteome 2005 4 1361 1370 10.1021/pr0500511 16083288

[B49] Pappin DJ Peptide mass fingerprinting using MALDI-TOF mass spectrometry Methods Mol Biol 2003 211 211 219 12489433 10.1385/1-59259-342-9:211

[B50] Lauderback CM Hackett JM Huang FF Keller JN Szweda LI Markesbery WR Butterfield DA The glial glutamate transporter, GLT-1, is oxidatively modified by 4-hydroxy-2-nonenal in the Alzheimer's disease brain: the role of Abeta1-42 J Neurochem 2001 78 413 416 10.1046/j.1471-4159.2001.00451.x 11461977

[B51] Jessop CE Bulleid NJ Glutathione directly reduces an oxidoreductase in the endoplasmic reticulum of mammalian cells J Biol Chem 2004 279 55341 55347 10.1074/jbc.M411409200 15507438

[B52] Kim-Han JS O'Malley KL Cell stress induced by parkynsonian mimetic, 6-hydroxydopamine, is concurrent with oxidation of the chaperone, ERp57, and aggresome formation Antioxid Redox Signal 2007 9 2255 2264 10.1089/ars.2007.1791 17848102

