# Supplementary material for: Effects of UVB-induced oxidative stress on protein expression and specific protein oxidation in normal human epithelial keratinocytes: a proteomic approach
Source: Proteome Sci. 2010 Mar 18;8:13. doi: 10.1186/1477-5956-8-13 (PMC3161386; doi:10.1186/1477-5956-8-13)
Supplement: Additional file 1 — Identification of UVB sub-toxic dose. Data provided describe the UVB Dose-response and time-course tissue culture toxic effect; Description of methods, results, comments, figures and references are provided. [file 1477-5956-8-13-S1.DOC]

Identification of UVB sub-toxic dose

The dose of UVB to be used was chosen through cytotoxic effect dose response and time course experiments.

Cytotoxic effect dose response experiment:

UBV toxicity on NEHK was evaluated in comparison with other continuous epithelial cell lines, i.e.: HK-168; HPK-Ia; Siha; C-33a. HK-168 and HPK-Ia are cell lines derived from normal human epithelial keratinocytes (NHEK) transfected with whole HPV-16 genome; these lines are not tumorigenic in nude mice. SiHa and C-33A are cell lines derived from invasive cervical carcinomas. SiHa cells harbour an integrated, single copy of HPV-16 genome; C-33A cells were derived from a cervical carcinoma devoid of any known HPV sequence.

Epithelial cells were seeded at 40,000 cell/cm^2^  in a 96 well plate. After overnight incubation medium was removed, the monolayers were irradiated, re-fed with fresh medium and placed back in the incubator. Cells viability was evaluated at 24 h post irradiation. Cell viability was determined by the MTT reduction method (Gerlier and Thomasset, 1986) with minor modifications as previously described (De Marco and Marcante, 1993). Figure S1 shows that in transformed and neoplastic keratinocytes, as well as in NHEK, UVB doses ranging from 10 to 80 J/m^2^ induced levels of cell toxicity ranging from slight to severe and that a 20 J/m^2^ dose induced in NHEK cells an intermediate level of toxicity.

Cytotoxic effect time course experiment.

Cells were plated at 30,000 cells/cm^2^ in a 96 well plate. After overnight incubation the medium was removed, the monolayers were irradiated, re-fed with fresh medium and placed back in the incubator. At 24, 48 and 72 h post irradiation cell viability was evaluated with MTT. Figure S2 shows that a decrease of cell growth is already evident in irradiated cultures at 24 h post irradiation. At later times (48 and 72 h) divergence from non irradiated cells further increases and the growth arrest become evident. We have already reported that such a growth arrest is accompanied by a fair induction of apoptotic death (De Marco et al 2007).

Thus a 20 J/m^2^ UVB dose, clearly adequate for cell damage, was chosen for subsequent experiments. These experiments on protein oxidation were performed at 5 hours post irradiation. They report on the state of proteome during the reaction phase and are not biased by late degenerative phenomena.

In some papers a sharply higher UVB dose is reported. Difference in dose used may depend on many factors including: the cell type used, the kind of effect one is looking for; the experimental design, the parameters measured, the efficiency of lamps; chamber geometry, protocol of administration and, above all, the used methods for the evaluation of administered dose. In many reports indeed cells are exposed while covered by PBS or medium. Thus the incident energy on cells is calculated with the help of a correction factor estimating the shield power of the liquid phase. In our work the keratinocytes are exposed without medium (as it occurs for human skin keratinocytes) and cautions were taken to avoid cell over-heating (see methods). We believe these aspects may explain why interesting biological (dose-response and growth curve) and biochemical results were obtained with an apparently low dose of UVB.

References

De Marco, F., Marcante, M.L., 1993. HPV-16 E6-E7 differential transcription induced in Siha

cervical cancer cell line by interferons. J. Biol. Regul. Homeost. Agents 7, 15-21.

De Marco F**,** Perluigi M, Foppoli C, Blarzino C, Cini C, Coccia R, Venuti A. (2007). UVB

irradiation down-regulates HPV-16 RNA expression: Implications for malignant progression of

transformed cells. Virus Res.130(1-2):249-259

Gerlier, D,. Thomassset, N., 1986. Use of MTT colorimetric assay to measure cell activation. J

Immunol Methods 94, 5763-5769.

Figures legend

Figure S1: UVB TOXICITY DOSE RESPONSE CURVE.

In several transformed keratinocytes, as well as in NHEK, UVB doses ranging from 10 to 80 J/m^2^ induced levels of cell toxicity ranging from slight to severe.

Cells were irradiated with various UVB doses. 24 h after irradiation, cell viability was determined by the MTT method. Results are given as percentage of respective controls (i.e. untreated cells of each cell line) and are the mean ± S.D. of eight independent replicas of a representative experiment in a set of five.

Figure S2: UVB TOXICITY TIME COURSE

A decrease of cell growth is already evident in irradiated cultures at 24 h post irradiation. At later times (48 and 72 h) divergence from non irradiated cells further increases and the growth arrest become evident.

Results are and are the mean ± S.D. of eight independent replicas of a representative experiment in a set of five.

Figure S1

UVB TOXICITY DOSE RESPONSE CURVE.

Figure S2

UVB TOXICITY TIME COURSE
